# Identification of She3 as an SCF^Grr1^ Substrate in Budding Yeast

**DOI:** 10.1371/journal.pone.0048020

**Published:** 2012-10-29

**Authors:** Ruiwen Wang, Mark J. Solomon

**Affiliations:** Yale University, Department of Molecular Biophysics and Biochemistry, New Haven, Connecticut, United States of America; University of Minnesota, United States of America

## Abstract

The highly orchestrated progression of the cell cycle depends on the degradation of many regulatory proteins at different cell cycle stages. One of the key cell cycle ubiquitin ligases is the Skp1-cullin-F-box (SCF) complex. Acting in concert with the substrate-binding F-box protein Grr1, SCF^Grr1^ promotes the degradation of cell cycle regulators as well as various metabolic enzymes. Using a yeast two-hybrid assay with a Grr1 derivative as the bait, we identified She3, which is an adaptor protein in the asymmetric mRNA transport system, as a novel Grr1 substrate. We generated stabilized She3 mutants, which no longer bound to Grr1, and found that the degradation of She3 is not required for regulating asymmetric mRNA transport. However, She3 stabilization leads to slower growth compared to wild-type cells in a co-culture assay, demonstrating that the degradation of She3 by Grr1 is required for optimal cell growth.

## Introduction

Ubiquitin-dependent protein degradation is important for the regulation of many cellular activities, including cell growth, morphogenesis, and cell cycle progression. The attachment of ubiquitin to lysine residues of target proteins is catalyzed by the sequential action of an E1 ubiquitin-activating enzyme, an E2 ubiquitin-conjugating enzyme, and an E3 ubiquitin ligase. The tagged protein is then recognized and degraded by the 26S proteasome. Two classes of ubiquitin ligases have been intensively investigated for their roles in cell cycle progression [1]: the anaphase-promoting complex/cyclosome (APC/C) and the Skp1-cullin-F-box protein complex (SCF).

The F-box protein in SCF complexes is the subunit responsible for recognizing substrates, usually in a phosphorylation dependent manner. Of the eleven F-box proteins identified in budding yeast, only three (Cdc4, Grr1 and Met30) have been found to form SCF complexes and to participate in substrate ubiquitination [2]–[4]. In addition to an F-box motif, which mediates binding to Skp1 within the SCF complex [5], these proteins also contain a substrate-binding region consisting of WD (Trp-Asp) repeats or LRR (leucine-rich) repeats [2]. These repeats specifically recognize the phosphorylated motif (phospho-degron) within substrates [6]. SCF^Cdc4^ mediates the degradation of cell cycle regulators such as the Cdc28 inhibitors Sic1 [7], [8] and Far1 [9] and of the replication protein Cdc6 [10]. SCF^Met30^ targets the Cdc28 inhibitory protein kinase Swe1 [11] and the transcription factor Met4 [12]. Both Met30 and Cdc4 contain WD repeats.

Grr1 is an 1151 amino acid, non-essential F-box protein utilizing an LRR region for substrate recognition [6], [13]. It was identified as a central component in glucose-induced signal transduction [14]. When glucose is abundant, the degradation of Mth1 via Grr1 leads to the induction of the glucose transporter Hxt1, thus increasing glucose entry into cells [15]. Grr1 is also responsible for the ubiquitination and degradation of several metabolic enzymes and proteins involved in glycolysis and amino-acid biosynthesis, such as His4 and Pfk27 [16]. The absence of Grr1 causes several metabolic defects including reduced fitness in various growth conditions and auxotrophy for aromatic amino acids [7], [14]. In addition to its metabolic functions, Grr1 also regulates cell cycle progression by targeting the G1 cyclins Cln1 and Cln2 [17], the cytoskeletal regulator Gic2 [18], and the cytokinesis protein Hof1 [19]. The degradation of Cln1 and Cln2 is required for a proper transition from G1 phase to S phase, whereas the degradation of Gic2 and Hof1 is required for efficient bud emergence and cell separation during cytokinesis, respectively. Although Grr1 is not essential, its deletion causes severely retarded growth [13], presumably resulting from the stabilization of multiple cell cycle regulators.

Haploid budding yeast cells exist in either of two mating types, a or α, determined by whether the *MATa* or the *MATα* cassette is present at the mating type locus [20]. After mitotic divisions, mother cells usually switch their mating type, whereas daughter cells do not [21]. The interconversion between *MATa* and *MATα* is initiated by the *HO* endonuclease, whose expression is restricted to mother cells. This uneven distribution of *HO* activity is caused by the asymmetric localization of a transcriptional repressor, Ash1 [22]–[24], to daughter cells. Ash1 suppresses the transcription of *HO* in daughter cells, thereby limiting *HO* expression and enabling the mating type switch to occur only in mother cells. The asymmetric distribution of Ash1 is caused by the transport of *ASH1* mRNA to daughter cells via a protein complex composed of Myo4 [23], [25], a myosin motor protein, She2 [26], an RNA binding protein that binds *ASH1* mRNA, and She3 [23], an adapter protein that links She2 and Myo4. In addition to Ash1, this protein complex is also responsible for the selective transport of a number of other mRNAs from mother cells to daughter cells [27].

Due to the slow growth of cells lacking Grr1, we sought to identify novel SCF^Grr1^ substrates that might regulate cell growth. Using a yeast two-hybrid assay to identify Grr1-binding proteins, we found that She3 is an SCF^Grr1^ substrate. We identified two She3 residues that are critical for its instability and its interaction with Grr1. Although She3 is necessary for maintaining normal Ash1 levels, its degradation is not required for the asymmetric localization of Ash1 to daughter cells. However, using a sensitive co-culture assay, we found that stabilization of She3 reduced cell fitness compared to wild-type cells, thus demonstrating that She3 degradation by SCF^Grr1^ contributes to optimal cell growth.

## Results

### Identification of She3 as a novel SCF^Grr1^ substrate

**Figure 1:**
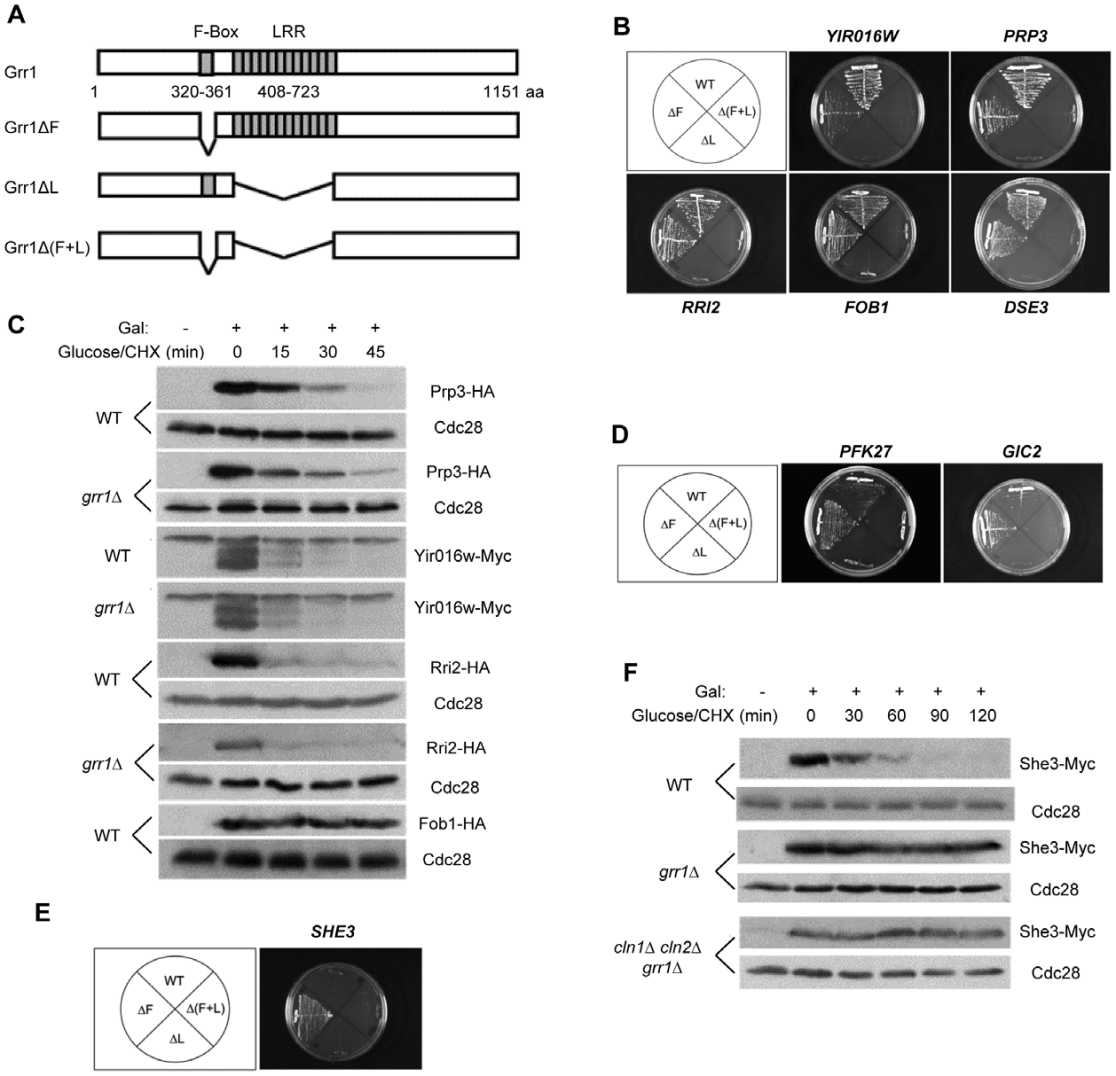
Identification of She3 as an SCF^Grr1^ substrate. (A) Structure of Grr1 and the deletion mutants used in this study. (B) Interactions of Grr1 mutants with several proteins that appear not to be SCF^Grr1^ substrates. (C) Degradation of Prp3, Yir016w, Rri2 and Fob1 in the indicated strains. WT: YJB15; *grr1Δ*: DOY805. Proteins with the indicated tags were expressed from a Gal promoter for 50 min. Samples were collected at the indicated times after adding cycloheximide and glucose to the cultures and analyzed by immunoblotting against the epitope tag. (* indicates a nonspecific band recognized by the anti-Myc antibody.) Proteins were expressed from the following plasmids (Supplemental Table 1): pRW0511083 (Prp3), pRW0511085 (Yir016w), pRW1022081 (Rri2), and pRW1103083 (Fob1). (D) Interaction patterns of Grr1 substrates (Pfk27 and Gic2) with the Grr1 mutants. (E) Interaction pattern of She3 with the Grr1 mutants. (F) The degradation of She3 was analyzed in wild-type cells (YJB15), *grr1Δ* cells (DOY805), and *grr1Δ cln1Δ cln2Δ* cells (DOY855). Cdc28 was used as a loading control in (C) and (F).

We used a yeast two-hybrid screen to identify potential SCF^Grr1^ substrates based on their interaction with wild-type Grr1 but not with a derivative of Grr1 lacking its leucine-rich repeats (LRR), the region known to bind substrates. We initially used full-length Grr1 as the bait. We tested interacting proteins for interactions with Grr1 mutants lacking the F box (Grr1ΔF), the LRR (Grr1ΔL), or both sequences (Grr1Δ(F+L)) (Fig. 1A). We expected that substrates would interact with Grr1 but not with Grr1ΔL and Grr1Δ(F+L). Out of 120 Grr1-interacting proteins analyzed, five met these criteria (Fig. 1B). However, the half-lives of Prp3, Yir016w and Rri2 were similar in wild-type and *grr1*Δ cells, whereas Fob1 was a stable protein (Fig. 1C). We could not detect galactose-induced expression of Dse3 in either wild-type or *grr1*Δ cells, indicating that it is rapidly turned over. These results indicate that none of these proteins is likely to be an SCF^Grr1^ substrate, although we cannot exclude the less likely possibilities that they could be ubiquitinated by SCF^Grr1^ but not subsequently degraded or that protein overexpression may have masked Grr1-dependent instability. These results also suggest that while the LRR of Grr1 is required for association with substrates, it may also have additional functions mediated by interactions with non-substrate proteins.

We wondered whether the failure to identify Grr1 substrates using wild-type Grr1 as the bait protein could be due to degradation of substrates by the overexpressed Grr1 from the two-hybrid vector. We tested this hypothesis by analyzing the interactions of two known Grr1 substrates, Gic2 and Pfk27 with wild-type and mutant forms of Grr1 (Fig. 1D). Both proteins interacted much more strongly with Grr1ΔF (which cannot bind Skp1 or promote protein degradation) than with wild-type Grr1, thus supporting this hypothesis. Importantly, neither protein interacted with Grr1 lacking its LRR.

Based on the above findings, we performed another screen for Grr1-binding proteins, this time using Grr1ΔF as the bait protein. Out of 120 clones analyzed, only one, containing She3, interacted with Grr1ΔF but not with Grr1ΔL or Grr1Δ(F+L) (Fig. 1E). Note that She3 failed to interact with wild-type Grr1, explaining why it was not identified in our initial screen. She3 was found to be a relatively unstable protein in asynchronous cells, with a half-life of about 25 minutes, whereas She3 was stable in *grr1*Δ cells (Fig. 1F). Since SCF^Grr1^ also promotes the degradation of the G1 regulators Cln1 and Cln2, we were concerned that stabilization of She3 might be an indirect effect following the stabilization of Cln1 and Cln2. This possibility was ruled out by the observation that She3 was also stable in cells deleted for *GRR1*, *CLN1* and *CLN2* (Fig. 1F). Thus, these results indicate that Grr1 regulates She3 stability.

### Myo4 and She2 play minimal roles in regulating She3 stability

**Figure 2:**
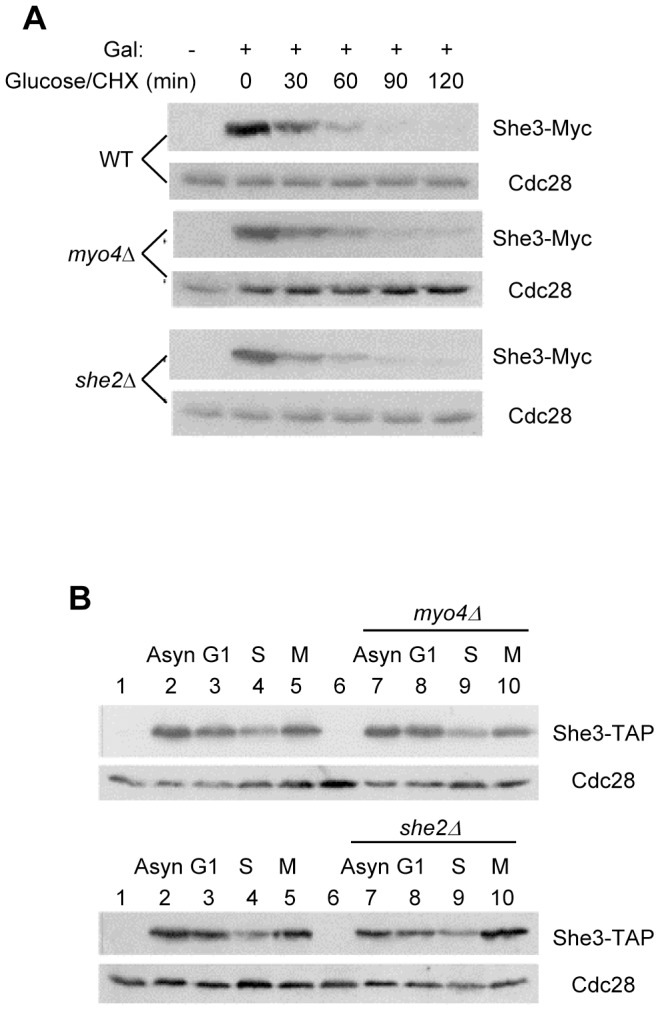
Neither Myo4 nor She2 affects She3 stability. (A) She3 degradation was analyzed in wild-type (YRW0523091), *myo4Δ* (YRW0927091) and *she2Δ* (YRW0531091) cells expressing TAP-tagged She3. (B) She3 levels were analyzed in the same wild-type, *myo4Δ* and *she2Δ* strains arrested in G1, S and M phases or in asynchronous cells (lanes 2 and 7). Lanes 1 and 6 are samples from control cells not expressing She3-TAP. She3-TAP was detected via its TAP tag. Cdc28 was used as a loading control.

She3 binds stably to the type V myosin motor protein Myo4 via its N-terminal tail and to the RNA-binding protein She2 through its C-terminal region. We wondered whether complex formation might regulate the stability of She3. It was previously found that the level of Myo4 is not affected by the presence of either She3 or She2 [28]. We found that deletion of *SHE2* or *MYO4* had little effect on She3 stability (Fig. 2A) or on its levels during various phases of the cell cycle (Fig. 2B). Interestingly, it appears that the amount of She3 is lower in S phase-arrested cells than in the other cell cycle phases; the underlying reason for this difference remains to be investigated. This effect could result from cell cycle fluctuations in She3 levels, or from a reduction in She3 level during the DNA replication checkpoint caused by hydroxyurea treatment.

### Identification of She3 mutants that are resistant to Grr1-mediated degradation

**Figure 3:**
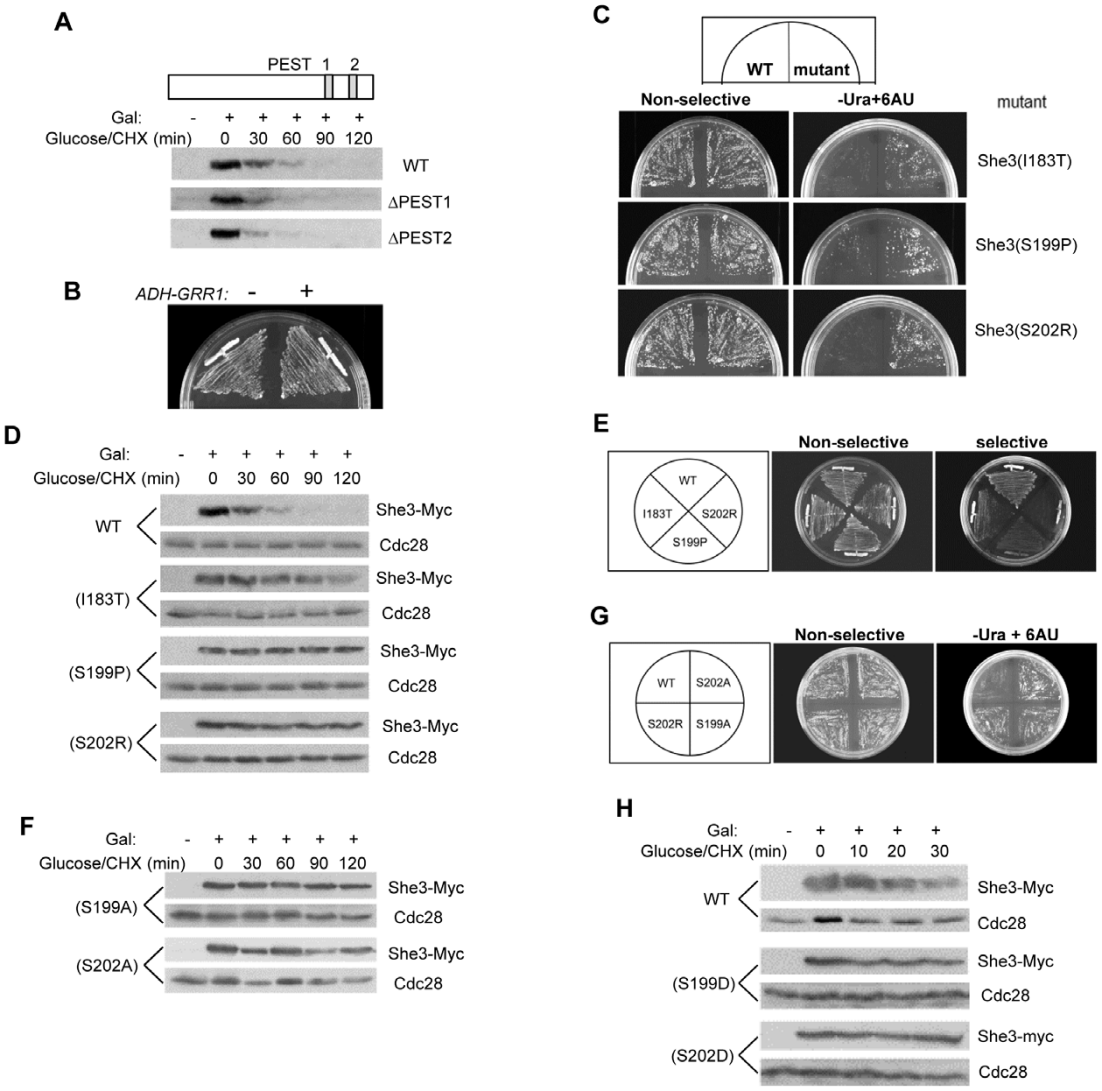
Identification of mutations that stabilize She3. (A) PEST motifs do not promote She3 degradation. Two potential PEST regions in She3 are shown schematically (residues 323–340 and 387–405). The degradation patterns of wild-type, ΔPEST1, and ΔPEST2 forms of She3 were compared. Strains used are YRW0129091 and YRW0222092. (B) Cells expressing *ADH-SHE3-URA3* with or without *ADH-GRR1-Myc* were tested for growth in selective minimal medium without uracil (CM-Ura-His). (C) Cells expressing wild-type and mutant forms of She3-Ura3 from the *ADH* promoter were tested for growth in the presence (left) or absence (right) of uracil. All plates lacked histidine to select for the *ADH-SHE3-URA3* plasmid. Plasmids used for transformation: pRW0416093 (WT), pRW0831098 (I183T), pRW0816093 (S199P) and pRW0816095 (S202R). Plates lacking uracil also contained 2.5 µg/ml 6-AU to inhibit Ura3 activity. (D) Degradation of wild-type and mutant forms of She3. Strains used: YRW0917091 (I183T), YRW0827092 (S199P) and YRW0827093 (S202R). (E) Interaction of wild-type and mutant She3 proteins with Grr1ΔF in the yeast two-hybrid assay. (F) Degradation of the indicated She3 mutant proteins. (G) Wild-type She3 and the indicated She3 mutants were tested for their ability to support cell growth as She3-Ura3 fusions proteins in selective medium as in (C). (H) Degradation of wild-type and mutant forms of She3. Strains used: YRW1005091 (S199D) and YRW1005094 (S202D). Cdc28 was used as a loading control in (D), (F) and (H).

In order to study the biological significance of She3 degradation by Grr1, it was important to identify stable She3 mutants. Several Grr1 substrates including Cln1, Cln2 and Hof1 contain functional PEST motifs [19], [29], which are frequently found in unstable proteins. Using the PEST-find program [30], we located two potential PEST motifs in the C-terminal region of She3. To test whether either of these motifs was important for She3 instability, we deleted each motif and determined the half-life of the resulting protein. The half-lives of the deletion mutants were similar to each other and to that of wild-type She3 (Fig. 3A). These results led us to search for other sequences involved in She3 degradation.

We next used a genetic screen to identify stabilized She3 mutants. For this screen, we fused She3 to Ura3 to produce a She3-Ura3 fusion protein. Our expectation was that cells expressing Grr1 would degrade this fusion protein, rendering cells unable to grow in the absence of uracil. However, we found that cells expressing She3-Ura3 grew efficiently on medium lacking uracil even when an additional copy of *GRR1* was introduced into the cells (Fig. 3B). To reduce Ura3 activity so that She3-Ura3 expression was no longer sufficient to support cell growth, we added 2.5 µg/ml of the Ura3 inhibitor 6-azauracil (6-AU) to the medium to suppress the growth of wild-type cells expressing She3-Ura3. She3 mutants were generated by subjecting the *SHE3* open reading frame to error prone PCR (EP-PCR). The PCR products were transformed into yeast cells together with a gapped vector bearing the *ADH* promoter and *URA3* with homology to the ends of the mutagenized *SHE3* EP-PCR products. Recombination *in vivo* reformed plasmids expressing She3-Ura3 fusion proteins under the control of the *ADH* promoter.

Several mutant clones were isolated that grew in the presence of 6-AU. The She3-Ura3 plasmids were recovered and sequenced to pinpoint the mutations within She3. When mutants contained multiple mutations, single mutations were generated and tested for their ability to confer growth on 6-AU medium. We identified three single mutations (I183T, S199P and S202R) that allowed better growth than the wild-type She3-Ura3 fusion protein (Fig. 3C). Of these, cells expressing She3 with the S199P and S202R mutations grew better than those with the S183T mutation (Supplemental Fig. 1).

We also tested the effects of these mutations on the stability of unfused She3 and on the interaction of She3 with Grr1 in the two-hybrid assay. The S199P and S202R mutations fully stabilized She3, whereas the S183T mutation incompletely stabilized it (Fig. 3D). The stabilizing effects of these mutations on She3 were paralleled by their disruption of the interaction with Grr1ΔF in the two-hybrid assay (Fig. 3E). Since the S199P and S202R mutations involve changes from potentially phosphorylatable serine residues to a non-charged proline or to a positively charged arginine, we decided to test the effects of charges at these positions. Proteins containing alanines at these positions were stable and the She3-Ura3 fusion proteins containing these mutations allowed better cell growth than wild-type She3-Ura3 (Figs. 3F and 3G). Interestingly, mutations of Ser199 and Ser202 to aspartate to mimic potential phosphorylation of the serines also resulted in stable proteins (Fig. 3H). Thus, the presence of serines or phosphoserines, but not the charges of residues 199 and 202, appears to be important for the degradation of She3.

### She3 degradation is not required for the asymmetric localization of Ash1

**Figure 4:**
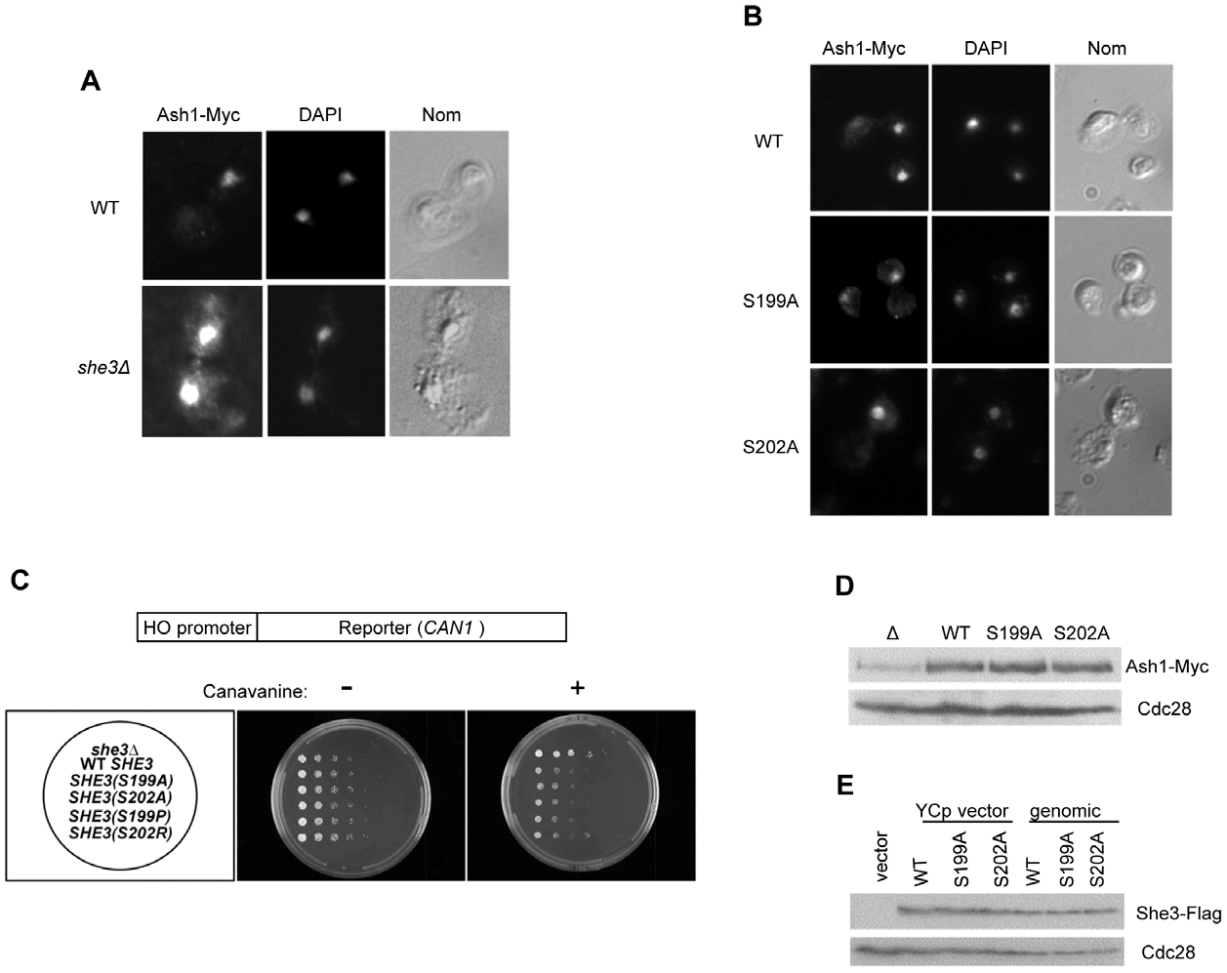
Stabilization of She3 has little effect on the asymmetric localization of Ash1. (A) Ash1-9xMyc localization was determined in strain K5552 (upper panels) or K5552 *she3Δ* (YRW0115101). Representative images of Ash1-9xMyc immunofluorescence, DAPI staining, and Nomarski optics are shown. (B) Both wild-type and stabilized forms of She3 restored the asymmetric localization of Ash1. Ash1-9xMyc localization was determined in K5552 *she3Δ* cells expressing wild-type or mutant She3 from the *SHE3* promoter on centromeric plasmids. Strains used: YRW0115101 (WT), YRW0121103 (S199A), YRW0121104 (S202A). (C) *HO* reporter assay for Ash1 localization. YLM923 cells (*she3Δ*) containing *HO-CAN1* expressed wild-type and stabilized forms of She3 from the *SHE3* promoter. Cells were serially diluted and grown on selective minimal medium (CM-Trp) with or without 0.03% canavanine. (D) Analysis of Ash1 levels in K5552 *she3Δ* cells expressing wild-type or mutant She3 proteins. Ash1 levels were determined by immunoblotting with anti-Myc antibodies. Strains used: YRW0115101(Δ), YRW0121102 (WT), YRW012103 (S199A) and YRW012104 (S202A). (E) The indicated forms of She3 were expressed from the endogenous *SHE3* chromosomal locus or from centromeric plasmids containing the *SHE3* promoter. Strains used in the last three lanes: YRW0314101 (WT), YRW0314103 (S199A) and YRW0314105 (S202A). The resulting levels of She3 were compared by immunoblotting with anti-Flag antibodies. Cdc28 was used as a loading control in (D) and (E).

She3 forms a complex with She2 and Myo4 to transport some mRNAs to daughter cells during cell division. The best studied such mRNA is the transcriptional repressor, Ash1. We tested the effects of She3 stabilization on *ASH1* mRNA distribution by analyzing the localization of Ash1 protein. As previously reported, Ash1 was mainly localized to daughters in wild-type cells whereas it was distributed to both mothers and daughters when *SHE3* was deleted (Fig. 4A). To test whether She3 degradation affected the asymmetric localization of Ash1, wild-type and stabilized forms of She3 were expressed from the endogenous *SHE3* promoter in *she3*Δ cells. Both wild-type and the stabilized She3 restored the asymmetric localization of Ash1 (Fig. 4B), indicating that She3 degradation is not required for this process.

We also used a genetic reporter assay to assess Ash1 localization in cells expressing wild-type or mutant She3 proteins [23], [31]. Ash1 is a transcriptional repressor that inhibits expression from the *HO* promoter. We expressed *CAN1* from the *HO* promoter. Can1 allows cells to take up more of the toxic arginine analog canavanine, which slows cell growth. The normal localization of Ash1 to daughter cells would allow Can1 expression in mother cells, resulting in their slow growth. In contrast, mis-localization of Ash1 to both mother and daughter cells (as in *she3*Δ cells) would enable cell growth in both cell types, resulting in more rapid overall growth. Both wild-type and stabilized forms of She3 slowed cell growth relative to *she3*Δ cells (Fig. 4C), further indicating that stabilization of She3 does not affect its ability to promote the asymmetric distribution of Ash1.

We also investigated whether wild-type or stabilized She3 affected the level of Ash1. Unexpectedly, we found that deletion of *SHE3* led to a reduction in Ash1 protein level, whereas there was little difference in Ash1 levels between cells expressing wild-type and stabilized forms of She3 (Fig. 4D).

In most of the above assays, She3 was expressed from centromeric plasmids. To exclude the possibility that expression from these plasmids may not reflect that from the endogenous *SHE3* locus, we compared protein levels of wild-type and stabilized forms of She3 expressed from the genomic *SHE3* locus and from plasmids. In all cases, protein levels were the same whether the gene was located on a plasmid or at the *SHE3* chromosomal location (Fig. 4E), indicating that plasmid-based expression faithfully mimics endogenous expression of She3. Interestingly, the overall levels of wild-type and mutant forms of She3 were very similar despite the stabilization of the She3 mutants, suggesting that a feedback mechanism may operate to maintain She3 protein levels despite variations in its stability.

### She3 stabilization reduces cell fitness

**Figure 5:**
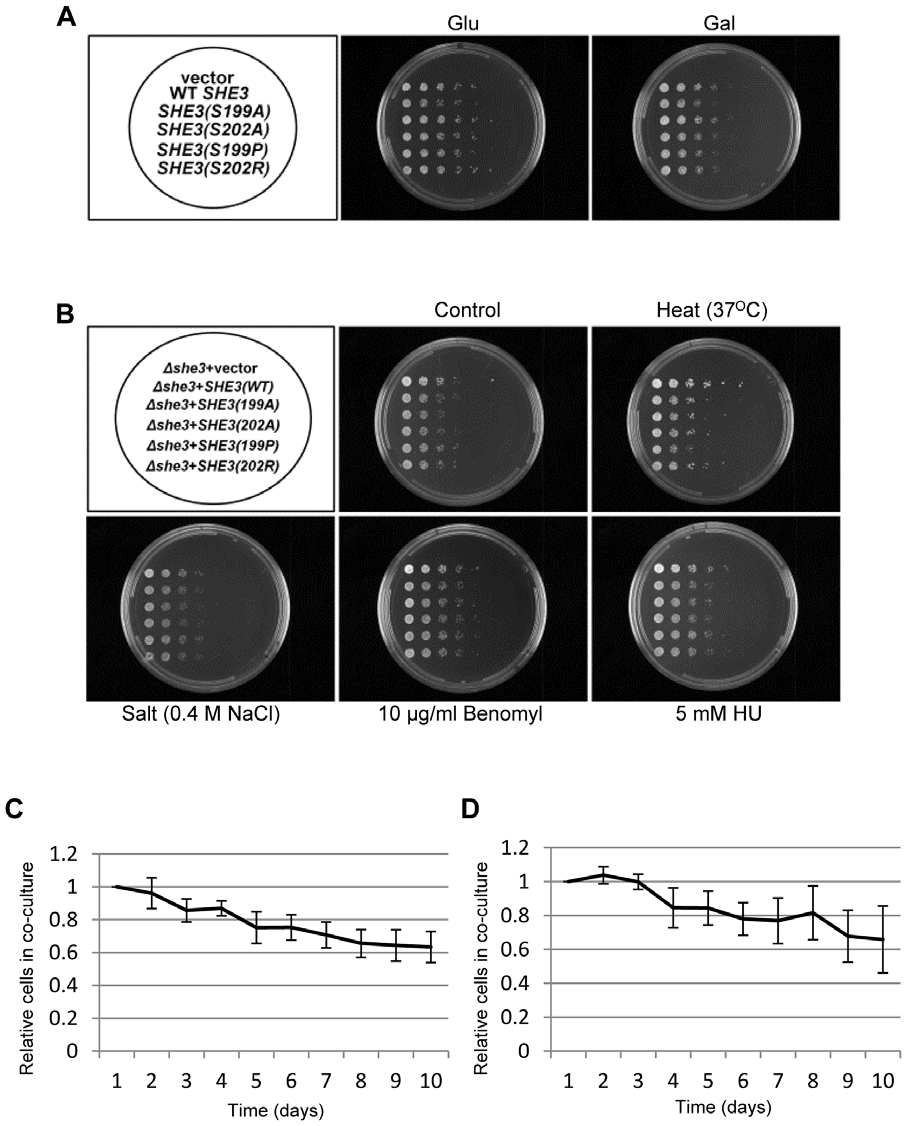
She3 degradation is required for optimal cell growth. (A) Wild-type and stabilized forms of She3 under the control of a *GAL* promoter were integrated into YJB15 cells at the *LEU2* locus. The resulting cells were serially diluted onto glucose- or galactose-containing selective minimal medium (CM-Leu) to determine the effects of She3 overexpression on cell growth. Strains used: YRW1127081 (WT), YRW0827092 (S199P), YRW0827093 (S202R), YRW1011092 (S199A) and YRW1011093 (S202A). (B) Wild-type and stabilized forms of She3 under the control of its own promoter were expressed in YJB15 *she3Δ* cells (YRW0517093). Plasmids used for transformation: pRW0115101 (WT), pRW0114101 (S199A), pRW0114103 (S199P), pRW0114105 (S202R) and pRW1221094 (S202A). Cells were grown in selective minimal medium (CM-Trp) with the indicated stress conditions. (C) YJB15 cells expressing wild-type She3-Flag from the endogenous *SHE3* locus (and containing a linked *TRP1* marker) (YRW0526112) were grown together with similar cells expressing She3 (S202A)-Flag (with a linked *LEU2* marker) (YRW0523113). The co-culture was diluted 1000-fold each day. The fraction of She3 (S202A)-Flag cells contained in the population was determined daily by plating on selective plates. (D) As in (C), but with the auxotrophic markers swapped (strains YRW0523111 and YRW0526113). Both (C) and (D) represent the averaged results of 5 independent experiments. Bars indicate the standard errors of the data.

We sought to determine whether stabilization of She3 affected cell growth. Overexpression of wild-type and mutant forms of She3 had no obvious effect on cell growth on plates (Fig. 5A). Similarly, we did not detect any obvious differences in growth under various stress conditions (Fig. 5B). We then turned to a sensitive co-culture experiment, which can detect differences in growth rate of well under 1% per generation. Cells expressing wild-type She3 or She3 (S202A) were genetically marked and grown together in a liquid culture. The culture was diluted each day and grown for ten days (about 100 generations). The fraction of mutant She3 cells in the culture was determined over time. Because the markers used might affect the relative growth of the strains, the markers were swapped and the co-culture experiment was repeated. Figs. 5C and 5D show the averaged results from five experiments with each marker configuration. We found that the proportion of cells expressing stabilized She3 (S202A) gradually declined by about one-third over ten days, indicating that these cells grew somewhat more slowly than wild-type cells. The apparent reduction in cell fitness was approximately 1.11% per generation.

## Discussion

We carried out a two-hybrid screen to identify proteins that bind to the substrate-binding LRR region of Grr1. One of these proteins, She3, was found to be a substrate of SCF^Grr1^ *in vivo*. We identified point mutations within She3 that disrupted its binding to Grr1, resulting in She3 stabilization. Although stabilization of She3 had no detectable effect on Ash1 localization, we found that it did result in a small reduction in cell fitness, indicating that She3 degradation plays a physiological role. This function, which may involve the daughter-cell localization of other mRNAs, remains to be identified. It should be noted that the assays we used to compare the activities of wild-type and stabilized forms of She3 are generally qualitative and perhaps more quantitative analysis will be required to reveal the subtle effects brought about by the stabilization. An important feature of our screen for Grr1-binding proteins was the use of Grr1ΔF to reduce substrate degradation during the interaction screen. This consideration should be applicable to the identification of substrates of other ubiquitin ligases.

It appears that the levels of She3 fluctuate, with the lowest level achieved during an S phase arrest. This fluctuation suggests that the role of She3 degradation by Grr1 may be limited to a small portion of the cell cycle or to the response to the DNA replication checkpoint. Interestingly, although *she3*Δ cells contain reduced levels of Ash1, cells with stabilized She3 have similar amounts of Ash1 as wild-type cells, likely because the levels of wild-type and mutant She3 are similar. How the cell maintains a constant level of She3 is currently unknown.

The LRR region of Grr1 is important for its interaction with substrates and its deletion results in cells morphologically similar to *grr1*Δ cells. We found a large number of proteins that could interact with Grr1 and Grr1ΔF as well as several proteins that interacted specifically with the LRR, but that appeared not to be Grr1 substrates. This finding raises the possibility that Grr1 may have additional regulatory functions besides targeting proteins for degradation. It is possible that non-substrate proteins that interact with the LRR may modulate Grr1 activity, perhaps by competing with substrates for binding to Grr1. Several such pseudosubstrate inhibitors have been found to regulate APC activity and at least one has been found to inhibit an SCF complex [32]–[35].

Interestingly, while our two-hybrid screen led to the identification of a novel Grr1 substrate, it failed to identify a number of known Grr1 substrates. As with any yeast two-hybrid screen, many verified interactors simply fail to produce a signal in the two-hybrid setup. Second, we only studied a limited number of Grr1-interacting proteins and the known substrates may have been present at a low abundance in the library we screened. Finally, it is possible that overexpression of some Grr1 substrates in the yeast two-hybrid vector is toxic, as we have observed in the case of Cln2.

Although SCF complexes generally recognize phosphodegrons, we do not yet know whether phosphorylation of She3 is required for its binding to Grr1. She3 contains numerous serine and threonine residues (93 residues from a total of 435 residues). Of these, a number have been found to be phosphorylated in several global phosphorylation analyses; these sites include residues 28, 217, 343, 348, 392 and 394 [36]–[39]. Two of the mutations identified in this study involve serines, but neither is among the known phosphorylation sites. Currently, we do not know whether any of the three sites at which we identified mutations is phosphorylated *in vivo*. Interestingly, the NetPhosYeast program [40] predicts that Ser-199 and Ser-202 could both be phosphorylated by casein kinase I. Although we found that mutation of these residues to Ala, Arg or Asp stabilized She3, it is still possible that phosphorylation of one or both of these sites affects She3 degradation since Asp, with a charge of −1, does not always fully mimic a phosphorylated amino acid, with a charge of −2. Further work will be required to address the potential role of phosphorylation in She3 degradation.

## Materials and Methods

### Plasmid and strain constructions

pAS2-*GRR1* was made by insertion of full-length *GRR1* into pAS2 between the *Nco*1 and *Bam*H1 sites, so that Grr1 was in frame with the Gal4 DNA binding domain sequence. pACTII-*SHE3* was made by insertion of *SHE3* into pACTII between the *Nco*1 and *Bam*H1 sites. The pAS2 (bait) and pACTII (prey) plasmids and the yeast cDNA library containing Gal4 activation domain fusions were gifts from Steven Elledge (Harvard Medical School, Boston, MA). *Gal-SHE3-Myc* was made by insertion of the *SHE3* coding sequence into YIp128 [41] containing a *Gal* promoter and three copies of the Myc-epitope tag. This plasmid (pRW1121083) was linearized with *Afl*II for integration at the *LEU2* locus. Similarly, *PRP3*, *YIR016W*, *RRI2*, *FOB1* and *DSE3* (mentioned in Fig. 1) were ligated into YIp128 to express proteins with three copies of the Myc- or HA-epitope tag at the C terminus. These constructs were used for protein half-life studies. Cells expressing TAP-tagged She3 (YRW0523091) were from the TAP-tagged yeast library described previously [42].

*ADH-SHE3-URA3-HA* was made by inserting *SHE3* into pRS313 containing *URA3-HA* under the control of the *ADH* promoter (pRW0416091) so that She3 was in frame with Ura3 (pRW0416093). To make a Flag-tagged version of She3 expressed from the *SHE3* promoter in YCp22 [41], we first cloned 500 base pairs upstream of the *SHE3* start codon into YCp22 containing three copies of the Flag epitope tag. The *SHE3* coding sequence was then placed between the promoter and the Flag tag (pRW0115101). To replace the endogenous copy of *SHE3* with Flag-tagged wild-type or mutant *SHE3*, a truncated version of *SHE3* beginning at base pair 318 of the coding sequence in frame with a C-terminal Flag tag was inserted into YIp204 (WT: pRW0310101), linearized with *Hpa*I within *SHE3*, and transformed into cells.

All the *SHE3* mutants were made using QuickChange mutagenesis and verified by sequencing. Detailed information on the mutations is available upon request. All of the studies were done in the YJB15 strain derived from W303-1A (*MATa ade2-1 his3-11,15 leu2-3,112 can1-100 ura3-1 trp1-1 ssd1-d*) [43] unless indicated otherwise. The *she3Δ*, *she2Δ* and *myo4Δ* strains were created by transforming cells with PCR products containing a NAT selection marker flanked by 5′ and 3′ sequences of the gene to be deleted [44]. The transformants were selected on plates containing 100 µg/ml nourseothricin. Deletions were verified by PCR using a primer downstream of the deleted gene and a primer internal to the NAT gene.

The strains and plasmids used in this study are listed in Table 1 and Supplemental Table 1, respectively. More information is available upon request.

**Table 1:** Strains used in this study.

Strain	Genotype	Source
YJB15	W303-1A *bar1Δ*	32
PJ69-4a	*MATa trp1-901 leu2-3,112 ura3-52 his3-200 gal4*Δ *gal80*Δ *LYS2::GAL1-HIS3 GAL2-ADE2 met2::GAL7-lacZ*	44
PJ69-4α	*MATα trp1-901 leu2-3,112 ura3-52 his3-200 gal4*Δ *gal80*Δ *LYS2::GAL1-HIS3 GAL2-ADE2 met2::GAL7-lacZ*	44
YLM923	*MATα ade2-1 leu2-3 trp1-1 ura3 HO-ADE2 HO-CAN1 she3::KAN*	31
K5552	*MATα ade2-1 trp1-1 can1-100 leu2-3,112 his3-11,15 ura3 ASH1-9xMyc*	25
DOY805	YJB15 *grr1::NAT*	33
DOY855	*MAT*a *grr1::LEU2 cln1*Δ *cln2::KAN his3*-200 *met25*Δ	This study
YRW0517081	YJB15 *LEU2:GAL-PRP3-HA*	This study
YRW0527082	DOY805 *LEU2:GAL-PRP3-HA*	This study
YRW0517082	YJB15 *LEU2:GAL-YIR016W-HA*	This study
YRW0519082	DOY805 *LEU2:GAL-YIR016W-HA*	This study
YRW1127081	YJB15 *LEU2:GAL-SHE3-Myc*	This study
YRW1215083	DOY805 *LEU2:GAL-SHE3-Myc*	This study
YRW0110091	KS499 *URA3:GAL-SHE3-Myc*	This study
YRW0523091	*MATa his3*Δ*1 leu2*Δ*0 met15*Δ*0 ura3*Δ*0 SHE3::SHE3-TAP* [HIS3]	
YRW0531091	YRW0523091 *she2::NAT*	This study
YRW0927091	YRW0523091 *myo4::NAT*	This study
YRW1110091	YJB15/pRS313-*ADH-SHE3-URA3-HA*	This study
YRW0917091	YJB15 *LEU2:GAL-SHE3(I183T)-Myc*	This study
YRW0827092	YJB15 *LEU2:GAL-SHE3(S199P)-Myc*	This study
YRW0827093	YJB15 *LEU2:GAL-SHE3(S202R)-Myc*	This study
YRW1011092	YJB15 *LEU2:GAL-SHE3(S199A)-Myc*	This study
YRW1011093	YJB15 *LEU2:GAL-SHE3(S202A)-Myc*	This study
YRW1005091	YJB15 *LEU2:GAL-SHE3(S199D)-Myc*	This study
YRW1005094	YJB15 *LEU2:GAL-SHE3(S202D)-Myc*	This study
YRW0129091	YJB15 LEU2:GAL-*SHE3(ΔPEST1)-Myc*	This study
YRW0222092	YJB15 LEU2:GAL-*SHE3(ΔPEST2)-Myc*	This study
YRW0121102	YRW0115101/YCp22-*SHE3pro-SHE3-Flag*	This study
YRW0121103	YRW0115101/YCp22-*SHE3pro-SHE3(S199A)-Flag*	This study
YRW0121104	YRW0115101/YCp22-*SHE3pro-SHE3(S202A)-Flag*	This study
YRW0121105	YRW0115101/YCp22-*SHE3pro-SHE3(S199P)-Flag*	This study
YRW0121106	YRW0115101/YCp22-*SHE3pro-SHE3(S202R)-Flag*	This study
YRW0314101	YRW0115101 *SHE3::SHE3-Flag* [YIp204]	This study
YRW0314103	YRW0115101 *SHE3::SHE3(S199A)-Flag* [YIp204]	This study
YRW0314105	YRW0115101 *SHE3::SHE3(S202A)-Flag* [YIp204]	This study
YRW1220092	YLM923/YCp22-*SHE3pro-SHE3-Flag*	This study
YRW1220093	YLM923/YCp22-*SHE3pro-SHE3(S199A)-Flag*	This study
YRW1220094	YLM923/YCp22-*SHE3pro-SHE3-Flag*	This study
YRW0523111	YJB15 *SHE3::SHE3-Flag* [YIp128]	This study
YRW0523113	YJB15 *SHE3::SHE3(S202A)-Flag* [YIp128]	This study
YRW0526112	YJB15 *SHE3::SHE3-Flag* [YIp204]	This study
YRW0526113	YJB15 *SHE3::SHE3(S202A)-Flag* [YIp204]	This study
YRW0115101	K5552 *she3::NAT*	This study
YRW0517093	YJB15 *she3::NAT*	This study
YRW0417091	YJB15/YIpac128-*ADH*-*GRR1*-Myc	This study

### Yeast two-hybrid analysis to identify Grr1 substrates

The yeast two-hybrid screens were performed as described previously [45] with minor modifications. Briefly, Grr1-interacting clones (preys) were identified by using pAS2-Grr1 or pAS2-Grr1ΔF as the bait in a yeast two-hybrid screen. Plasmids were isolated from these clones and retransformed into PJ69-4α cells [45]. These cells were then mated with PJ69-4a cells containing pAS2-Grr1 (WT), ΔF, ΔL or Δ(F+L) to generate diploid cells containing both the bait and prey plasmids for testing of interactions with the various Grr1 proteins. Interactions resulted in growth on selective plates (CM-Trp-Leu-His-Ade). The F-box and LRR regions deleted in the indicated forms of Grr1 span amino acids 320–361 and 408–723, respectively.

### Cell culture conditions

Yeast cells were grown in YPD or selective minimal medium as described previously [32]. For G1 arrest, cultures were grown to mid-exponential phase (OD_600_ of ∼0.3–0.5) followed by addition of 100 ng/ml α-factor (Sigma-Aldrich) for 2 hours. For S phase arrest, 100 mM hydroxyurea (Sigma-Aldrich) was added for 2 hours. For M phase arrest, cells were grown in medium containing 50 µg/ml benomyl (Dupont) for 2 hours.

For protein stability analysis, cultures were grown in YP-raffinose to exponential phase (OD_600_ of ∼0.3–0.5). 2% galactose was added and cultures were grown for 50 min to induce protein expression, followed by addition of 2% dextrose and 500 µg/ml of cycloheximide (Acros Organics). Cells were removed at the indicated times, washed once with H_2_O, and frozen in liquid nitrogen.

### Yeast extract preparation and immunoblot analysis

Cell pellets were suspended in three volumes of lysis buffer (6.7% sodium dodecyl sulfate (SDS), 75 mM Tris/Cl (pH 7.5), 27% glycerol, 100 mM dithiothreitol (DTT)). Cells were broken by shaking the suspension together with 0.45 g glass beads (∼300 µl volume) for 3 min in a bead beater, and then incubating at 95°C for 10 min. Glass beads and cell debris were removed by centrifugation at 14,000 rpm for 5 min. The supernatant was further clarified by centrifugation at 65,000 rpm in a TLA 100.2 rotor (Beckman) for 10 min at 15°C. Protein extracts were separated on a protein gel containing 8% polyacrylamide and transferred to an Immobilon-P membrane (Millipore). The membranes were incubated with 5% non-fat dried milk/TBST (10 mM Tris-Cl [pH 7.5], 150 mM NaCl, 0.05% Tween 20) for 2 h followed by incubation with primary and secondary antibodies. HA and Myc tags were detected with 12CA5 and 9E10 monoclonal antibodies (Covance Research Products), respectively. The Flag tag was detected with anti-Flag M2 monoclonal antibody (Sigma-Aldrich). The TAP tag was detected with Peroxidase Anti-Peroxidase rabbit antibody (PAP) (Sigma-Aldrich). Cdc28 was detected with anti-PSTAIRE antibodies [46]. Proteins were visualized by chemiluminescence (SuperSignal, Pierce).

### Error-prone PCR mutagenesis and isolation of stable She3 mutants

*SHE3* was mutagenized by error-prone PCR (EP-PCR) using pRW0416093 as a template and oligonucleotides MSO2731 and MSO2732 as primers. MSO2731 is a sense primer that anneals to the *ADH* promoter sequence upstream of *SHE3* in pRW0416093 and extends to the *SHE3* start codon: 5′- GTT CTC GTT CCC TTT CTT CCT TGT TTC TTT TTC TGC ACA ATA TTT CAA GCT ATA CCA AGC ATA CAA TCA ACT ATC TCA TAT ACA GGA TCC ATG -3′. MSO2732 is an antisense primer that anneals to the *URA3* sequence downstream of *SHE3*: 5′- GCA TGA TAT TAA ATA GCT TGG CAG CAA CAG GAC TAG GAT GAG TAG CAG CAC GTT CCT TAT ATG TAG CTT TCG ATC CGC CCG GCC GGT CGA C-3′. EP-PCR reaction conditions were as follows: 2 µg/ml template, 0.2 µM MSO2731, 0.2 µM MSO2732, 1 mM dTTP, 1 mM dCTP, 0.2 mM dATP, 0.2 mM dGTP, 6 mM MgCl_2_, 50 µM MnCl_2_, 1× PCR buffer (Roche), and 0.5 U/µl Taq polymerase. After 16 cycles of amplification, PCR reaction products were purified using the Qiagen PCR purification kit (Qiagen Inc.) and cotransformed into strain YRW0417091 together with gapped pRW0416091 vector, which was cut between the *ADH* promoter and the *URA3* sequence. Transformed cells were plated on medium containing 2.5 µg/ml 6-azauracil (AU) and lacking uracil to ensure correct recombination of PCR products with vector and production of She3-Ura3 fusion proteins. The clones that demonstrated enhanced growth after 36 hours were selected for further analysis (see below).

Plasmids were rescued and re-tested for their ability to support growth on 6-AU plates. The mutant *SHE3* genes were sequenced to identify the sites of mutations. If more than one mutation was identified, mutants with single mutations were generated and were tested as above to determine which could confer growth on 6-AU plates.

### Fluorescence microscopy

Indirect immunofluorescence was performed based on a modified protocol described previously [47]. Briefly, exponentially growing cells (OD_600_ of ∼0.3–0.6) were fixed with 3.7% formaldehyde for 1 hour followed by washing once with 0.1 M potassium phosphate (pH 7.5). The cells were then treated with 50 µg/ml Zymolyase-100T/0.3% β-mercaptoethanol/0.1 M potassium phosphate (pH 7.5) for 15–25 min at 30°C with continual monitoring. The cells were harvested and washed once with HS buffer (0.1 M Hepes (pH 7.4)/1 M Sorbitol) and permeabilized by incubation in HS/0.5% SDS for 5 min. Cells were harvested, washed once in HS buffer, and placed on polylysine-coated glass coverslips for 10 min. The coverslips were then blocked with PBS containing 5 mg/ml BSA and 0.02% Tween 20, incubated sequentially with primary antibodies (9E10 from Covance) in the same buffer, rhodamine-conjugated goat anti-mouse secondary antibodies (Jackson ImmunoResearch) and 1 µg/ml 4,6-diamidino-2-phenylindole (DAPI), and mounted on glass slides with Aquamount (Polyscience, Inc., Warrington, PA).

### Co-culture experiment

Similar to our previous analysis of APC substrates [48], cells expressing either wild-type or mutant (S202A) She3 with different auxotrophic markers (either *TRP1* and *LEU2*) were grown together in YPD supplemented with 50 µg/ml tryptophan and leucine at 23°C to OD_600_ ∼0.7. The co-cultures were diluted 1000-fold into fresh medium each day and propagated for 10 days. Aliquots were plated daily onto CM-Trp and CM-Leu plates to determine the fraction of SHE3-Flag (S202A) mutant cells in the population. Each co-culture was repeated 5 times and the results were averaged. The experiment was repeated after swapping the *LEU2* and *TRP1* markers.

The relative fitness of the mutant strain was defined as the fraction of a cell cycle that the strain underwent in the time the corresponding wild-type strain underwent one cell cycle. A close approximation to this value can be obtained from the formula: f = 1+ln(R)/(a^.^ln(2)), where f is the relative fitness of the mutant strain, R is the ratio of the number of mutant cells to the number of wild-type cells late in the growth of the co-culture (normalized to a ratio of 1 when the culture was started), and “a” is the number of generations undergone by the culture at the time of analysis.

## Supporting Information

Figure S1 **Growth of strains expressing mutant She3-Ura3 fusion proteins.** Cells expressing wild-type and mutant forms of She3-Ura3 from the *ADH* promoter were tested for growth in the presence (left) or absence (right) of uracil. All plates lacked histidine to select for the *ADH-SHE3-URA3* plasmid. Plates lacking uracil also contained 2.5 µg/ml 6-AU to inhibit Ura3 activity. Plasmids used for transformation: pRW0416093 (WT), pRW0831098 (I183T), pRW0816093 (S199P) and pRW0816095 (S202R).
(PDF)

Table S1 **Plasmids used in this study.** (All plasmids were constructed in this study.)
(PDF)
