# Supplementary figures and images for: Identification of She3 as an SCFGrr1 Substrate in Budding Yeast
Source: PLoS One. 2012 Oct 29;7(10):e48020. doi: 10.1371/journal.pone.0048020 (PMC3483296; doi:10.1371/journal.pone.0048020)

**Figure S1:**

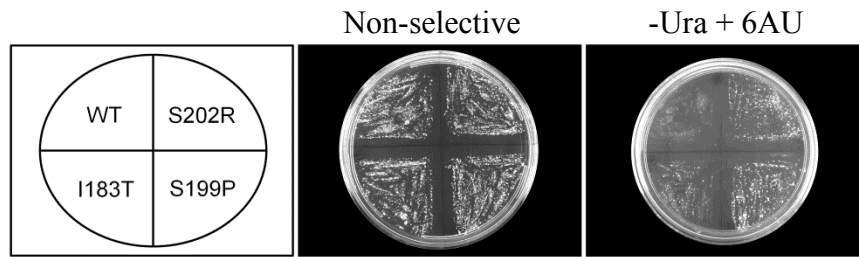

Supplement: Figure S1 — Growth of strains expressing mutant She3-Ura3 fusion proteins. Cells expressing wild-type and mutant forms of She3-Ura3 from the ADH promoter were tested for growth in the presence (left) or absence (right) of uracil. All plates lacked histidine to select for the ADH-SHE3-URA3 plasmid. Plates lacking uracil also contained 2.5 µg/ml 6-AU to inhibit Ura3 activity. Plasmids used for transformation: pRW0416093 (WT), pRW0831098 (I183T), pRW0816093 (S199P) and pRW0816095 (S202R). (PDF) [file pone.0048020.s001.pdf]
