# Supplementary material for: Identification of She3 as an SCFGrr1 Substrate in Budding Yeast
Source: PLoS One. 2012 Oct 29;7(10):e48020. doi: 10.1371/journal.pone.0048020 (PMC3483296; doi:10.1371/journal.pone.0048020)
Supplement: Table S1 — Plasmids used in this study. (All plasmids were constructed in this study.) (PDF) [file pone.0048020.s002.pdf]

Supplemental Data

Supplemental Table 1: Plasmids used in this study. (All plasmids were constructed in this study.)

Plasmid	Description
pRW0123081	pAS2-*GRR1*
pRW0409081	pAS2-*GRR1ΔL*
pRW0411081	pAS2-*GRR1ΔF*
pRW0422083	pAS2-*GRR1Δ(F+L)*
pRW0511083	YIp128-*GAL-PRP3*-HA
pRW0511085	YIp128-*GAL-YIR016W*-HA
pRW1022081	YIp128-*GAL-RRI2*-HA
pRW1103083	YIp128-*GAL-FOB1*-HA
pRW1121085	YIp128-*GAL-DSE3*-Myc
pRW1121083	YIp128-*GAL-SHE3*-Myc
pRW0106091	YIp211-*GAL-SHE3*-Myc
pRW0125091	YIp128-*GAL-SHE3(ΔPEST1)*-Myc
pRW0214092	YIp128-*GAL-SHE3(ΔPEST2)*-Myc
pRW0821091	YIp128-*GAL-SHE3(S199P)*-Myc
pRW0821093	YIp128-*GAL-SHE3(S202R)*-Myc
pRW0912097	YIp128-*GAL-SHE3(I183T)*-Myc
pRW0323093	YIp128-*ADH1-GRR1*-Myc
pRW0416091	pRS313-*ADH1-URA*-HA
pRW0416093	pRS313-*ADH1-SHE3-URA*-HA
pRW0811091	YCp22-*SHE3Pro-SHE3*-Myc
pRW0908091	YCp22-*SHE3Pro-SHE3(S199P)*-Myc
pRW0908092	YCp22-*SHE3Pro-SHE3(S202R)*-Myc
pRW0831098	pRS313- *ADH1-SHE3(I183T)-URA*-HA
pRW0816093	pRS313- *ADH1-SHE3(S199P)-URA*-HA
pRW0816095	pRS313- *ADH1-SHE3(S202R)-URA*-HA
pRW0929091	pRS313- *ADH1-SHE3(S199A)-URA*-HA
pRW0929094	pRS313- *ADH1-SHE3(S202A)-URA*-HA
pRW0128091	PACTII-*SHE3*
pRW0912091	PACTII-*SHE3(I183T)*
pRW0912094	PACTII-*SHE3(S199P)*
pRW0912095	PACTII-*SHE3(S202R)*
pRW0111101	PACTII-*SHE3(S199A)*
pRW0111103	PACTII-*SHE3(S202A)*
pRW1007095	PACTII-*PFK27*
pRW0115101	YCp22-*SHE3Pro-SHE3*-Flag
pRW0114101	YCp22-*SHE3Pro-SHE3(S199A)*-Flag
pRW0114103	YCp22-*SHE3Pro-SHE3(S199P)*-Flag
pRW0114105	YCp22-*SHE3Pro-SHE3(S202R)*-Flag
pRW1221094	YCp22-*SHE3Pro-SHE3(S202A)*-Flag
pRW0310101	YIp204-*SHE3*-Flag
pRW0309103	YIp204-*SHE3(S199A)*-Flag
pRW0310103	YIp204-*SHE3(S202A)*-Flag
